# Career duration in general practice: Evidence from Two Decades of Norwegian Registry Data

**DOI:** 10.1177/14034948251394639

**Published:** 2025-12-02

**Authors:** Øyvind Snilsberg, Johannes B. Tyrihjell, Geir Godager

**Affiliations:** 1 Department of Health Management and Health Economics, University of Oslo, Norway; 2 Oslo Economics, Oslo, Norway; 3 Health Services Research Unit, Akershus University Hospital, Norway

**Keywords:** Career duration, continuity of care, Norway

## Abstract

*Background:* Resignations among general practitioners (GPs) have been cited as a contributing factor to challenges in Norway’s GP scheme. *Aim:* To analyse trends in GP exit rates and examine whether there is evidence of systematic changes in these rates over time. *Design and setting:* Registry-based observational study of all GPs in Norway since the scheme’s implementation in 2001. *Method:* Exit rates were calculated for the entire study period and by year. Significant deviations were identified using 95% confidence intervals with Bonferroni corrections for multiple hypothesis testing. *Results:* GP exit rates declined until age 50 years and remained low until retirement at 67. This pattern was stable throughout the study period (2002–2022). Only two significant deviations occurred: higher-than-expected rates for 67-year-olds in 2007 and 41-year-olds in 2010. ***Conclusions:*** **GP exit rates remained stable over the study period, with no evidence suggesting increased exits as a major factor in the current challenges facing Norway’s GP scheme.**

## Introduction

Recruitment and retention of general practitioners (GPs) have become pressing topics in both research and policy [1,2]. While the Norwegian GP scheme has performed well on several quality indicators [3–5], recent years have brought substantial challenges. The number of citizens without a GP has reached historically high levels, and municipalities have been struggling to recruit new GPs [6]. GPs have reported excessive workloads [7] and declining job satisfaction [8]. Over the past decade, GPs have gradually reduced the size of their patient lists, decreasing the scheme’s overall capacity. Politicians [9], GPs [10], researchers [11] and consultancy reports [12] have all indicated that exit rates have increased in recent years. This alleged increase in exit rates has been linked to descriptions of growing workloads for GPs [13] and dissatisfaction with the government’s lack of prompt response to the ongoing crisis [9]. Whether the observed rise in the number of GPs leaving the profession reflects an increase in age-specific exit rates (the proportion of GPs of a given age leaving the profession) or simply demographic changes in the GP workforce – with more GPs reaching retirement age – is an important research question. Understanding the dynamics behind GP exits is crucial, as different interpretations can shape political responses. For instance, concerns about increasing exits have already influenced recent action plans for Norway’s primary care sector [14].

## Data

Norwegian primary care is organized as a list system, where citizens are registered on GP patient lists managed by their municipality. Municipalities are responsible for contracting GPs to ensure coverage for these lists, and GPs might manage multiple lists, even across municipalities. For this study, we use data from the FLO register, which contains quarterly observations of all GP patient lists from 2001, when the current GP scheme was introduced, to 2024. Since the scheme began in June 2001, our analysis focuses on the period from 2002 onward.

The FLO data allow us to classify GPs as having exited the profession if they end all GP contracts and remain inactive for more than eight consecutive quarters. This approach distinguishes between GPs who change offices or take temporary breaks and those who leave the profession entirely, thereby improving on previous research [12,15]. Since identifying exits requires GPs to remain inactive for more than eight quarters, data covering 2002 to 2024 enables us to calculate exit rates up to 2022.

Using quarterly data from 2002 to 2024, we first identified GP exits for the period 2002 to 2022. We then aggregated the data into one observation per GP per year, resulting in 94,337 GP-year observations. Each observation includes an indicator for whether the GP exited that year and for the GP’s age. Supplementary material Figure 2 online shows the number of GPs by age group per year. Since most GPs are aged 28 to 70 years, we truncated the age range to this interval. In the final step, we further aggregated the data into one observation per GP age group per year, covering 21 years (2002–2022) and 43 age groups (ages 28–70), for a total of 903 age–year observations. Each observation includes the number of GPs, the number of exits and the exit rates. Exit rates are calculated by dividing the number of exits by the number of GPs. The dataset and a Stata do-file are provided for replication.

## Methods

The aim of this analysis was to describe exit rates in Norwegian general practice from 2002 to 2022. We focus on three key metrics.

### 1. Exit rates by age

The share of GPs in each age group who exited during the study period, calculated as total exits in an age group divided by the total number of GPs in that group.

### 2. Exit rates by age and year

The share of GPs in each age group who exited each year, calculated by dividing exits by the number of GPs for each age and year. These are provided in the Supplementary material.

To investigate whether exit rates differ significantly from expected levels, we calculated Clopper–Pearson confidence intervals (CIs) and plotted them alongside the rates. To calculate a 95% confidence interval (CI), the lower bound was determined by finding the smallest number of exits, x , such that Pr(X≤x)≥0.025 , where X~Binomial(n,p) , with n as the number of GPs at risk and p as the overall exit rate for the age group. The lower bound was then calculated as *x/n*. For the upper bound, 0.025 was replaced with 0.975, and the corresponding x value was used. For example, in 2002, there were 21 GPs aged 28, and the exit rate among 28-year-olds was 0.079. Using Stata (or other software), the smallest x for which the cumulative probability is at least 0.975 is 4. Dividing 4 by 21 gives an upper Clopper–Pearson bound of approximately 0.190.

To account for multiple hypothesis testing, we also report Bonferroni corrected CIs. With 43 age groups and an initial alpha of 0.05, the correction adjusts alpha to 0.05/43, reducing the risk of false positives.

### 3. Exits by year

The total number of GPs leaving each year. Observed exits were compared with expected values, calculated by multiplying the number of GPs in each age group by their overall exit rate (from metric 1) and summing across age groups.

## Results

Table I and Figure 1 present the overall age-specific exit rates, showing the share of GPs who left the profession by age across the entire study period. The data show that younger GPs have a relatively higher exit rate, approximately 7% for the youngest age groups. This rate steadily declines with age, reaching its lowest point of around 2–3% among GPs in their 50s. As anticipated, exit rates rise sharply as GPs approach the retirement age of 67 years.

**Table I.** Exit rates by age in Norwegian general practice 2002–2022.

Truncated age	Observations	Observed to exit	Exit rate
28	647	51	0.079
29	815	55	0.067
30	1274	90	0.071
31	1700	103	0.061
32	2068	133	0.064
33	2311	128	0.055
34	2528	137	0.054
35	2677	135	0.050
36	2847	137	0.048
37	2898	129	0.045
38	2895	133	0.046
39	2902	115	0.040
40	2876	117	0.041
41	2843	118	0.042
42	2813	108	0.038
43	2721	107	0.039
44	2661	86	0.032
45	2600	96	0.037
46	2589	102	0.039
47	2564	75	0.029
48	2572	85	0.033
49	2615	84	0.032
50	2594	70	0.027
51	2577	71	0.028
52	2589	64	0.025
53	2558	64	0.025
54	2539	63	0.025
55	2514	67	0.027
56	2486	80	0.032
57	2430	76	0.031
58	2401	60	0.025
59	2355	61	0.026
60	2275	64	0.028
61	2187	66	0.030
62	2090	82	0.039
63	1960	89	0.045
64	1828	105	0.057
65	1658	139	0.084
66	1445	153	0.106
67	1218	348	0.286
68	804	174	0.216
69	582	128	0.220
70	831	367	0.442

**Figure 1.**
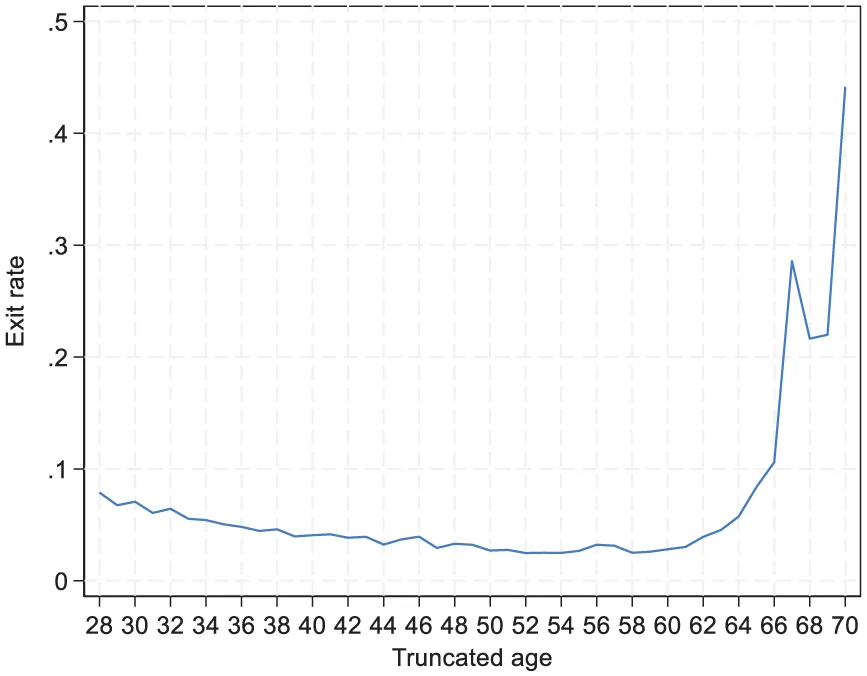
Exit rates by age in Norwegian general practice 2002–2022.

We next examined exit rates by age and year to explore trends over time and conduct hypothesis testing. Figure 2 presents exit rates for the first and last years of the study period, as well as for 2007 and 2010. Dark grey CIs represent unadjusted intervals, while light grey CIs reflect Bonferroni-corrected intervals. Age-specific exit rates by year for the period 2002–2022 are available in Supplementary Table S1 and Supplementary Figure S1. The years 2007 and 2010 are highlighted because they exhibit the only two statistically significant deviations from expected rates: for 67-year-olds in 2007 and 41-year-olds in 2010, both marked with red circles (Figure 2).

**Figure 2.**
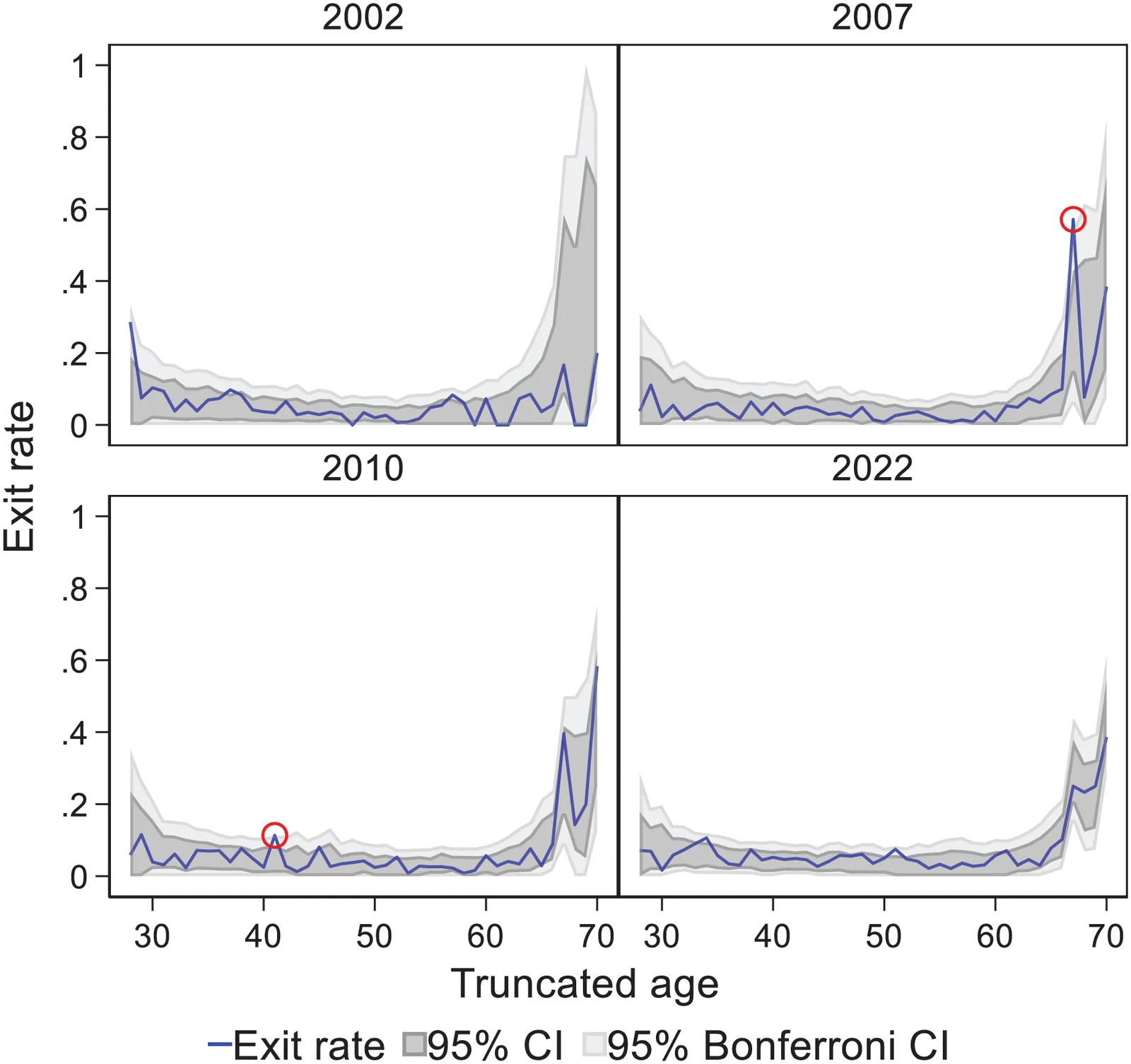
Exit rates by age and year in Norwegian general practice 2002, 2007, 2010 and 2022. CI: confidence interval

Finally, we compared the observed and expected number of GP exits for each year. Expected exits were calculated by multiplying the number of GPs in each age group by their overall exit rate and summing across all age groups. Table II and Figure 3 present a year-by-year comparison of observed and predicted exit counts.

**Table II.** Observed and predicted number of exits in Norwegian general practice 2002–2022.

Year	Observed	Predicted
2002	166	155
2003	173	159
2004	170	162
2005	191	163
2006	154	165
2007	167	172
2008	170	181
2009	158	189
2010	213	199
2011	187	208
2012	202	217
2013	237	229
2014	206	239
2015	236	250
2016	264	260
2017	268	272
2018	296	281
2019	295	290
2020	291	299
2021	318	303
2022	353	312

**Figure 3.**
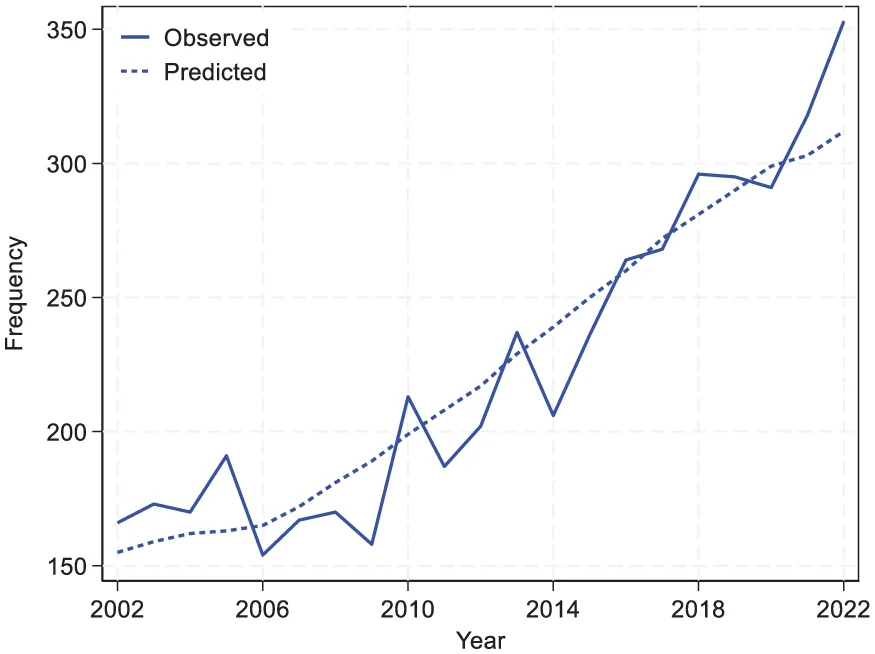
Observed and predicted number of exits in Norwegian general practice 2002–2022.

## Discussion

### Summary

This study examines age-specific exit rates in Norwegian general practice from 2002 to 2022 to assess their role in the current challenges facing the GP scheme. The findings show that age-specific exit rates have remained stable throughout the period, which challenges the view that a diminished attractiveness of a career in general practice has contributed to the recent capacity issues. This stability suggests that other factors, such as a rising number of GPs nearing retirement, might be more significant contributors to the current challenges.

### Strengths and limitations

A key strength of this study is its comprehensive national coverage. It includes all Norwegian GPs over a 21-year period and uses detailed registry data to identify long-term exits and distinguish them from relocations or temporary absences. The extended timeframe makes it possible to examine trends in the composition and stability of the GP workforce. That said, the analysis has limitations. First, our definition of exit, eight consecutive quarters without a GP contract, is intended to be cautious. This helps reduce the risk of misclassifying short-term absences as permanent exits but might exclude GPs who return after a longer break. It also means that exits from 2023 onwards are not captured. Second, the comparison between observed and predicted exits relies on the assumption that exit rates are stable over time. While the data support this, the prediction model is intentionally simple and intended to illustrate how stable and predictable GP exits have been historically. It might not capture short-term disruptions such as new policies or the COVID-19 pandemic, which could affect GP behaviour in ways that differ from past trends. Third, the analysis is descriptive and does not explore reasons for leaving, such as retirement, burnout or career change. Nor does it capture reduced availability among GPs who remain under contract but reduce their workload – changes that also affect capacity. Finally, the analysis does not account for variation across subgroups such as region, gender or practice type, which might influence both exit behaviour and local workforce pressures.

### Comparison with existing literature

Previous studies have highlighted increasing workload and declining job satisfaction among GPs in Norway and internationally [16–18]. Research points to administrative burdens, long working hours and poor work–life balance as factors associated with reduced career satisfaction and intentions to leave. While we do not measure satisfaction directly, the stability of age-specific exit rates challenges the assumption that large numbers of GPs are leaving owing to dissatisfaction [19,20]. This has broader relevance: in other high-income countries, GP attrition is a common concern, often based on surveys, anecdotal reports and observed workload increases. Although such concerns are also linked to an ageing workforce and recruitment difficulties, exits are often cited as a problem. The Norwegian case provides a useful counterpoint, showing that exit rates have remained stable despite growing concerns. At the same time, exits remain politically sensitive, and this study contributes by examining their actual role in ongoing capacity issues. The findings suggest that attrition should be understood in the context of broader demographic trends.

### Implications for practice

Our results illustrate that it is important to distinguish between the total number of annual exits (which has indeed increased over time) and age-specific exit rates (which remained stable). The increasing total exits observed in recent years (Figure 3) appear to be a consequence of demographic changes in the GP workforce rather than increasing attrition among specific age groups. The stability of age-specific exit rates indicates that capacity challenges in Norwegian general practice are not driven by more GPs leaving before retirement. Instead, these challenges reflect two broader developments: more GPs are reaching retirement age, and the gap between demand and service capacity is growing.

If retention issues were a primary driver of the current GP shortage, we would expect to see increases in age-specific exit rates over time. However, our data show no such trend. The data provide no evidence that GP retention has recently become a more pressing concern that requires immediate policy attention. Therefore, workforce planning may instead benefit from focusing on the demographic composition of the GP workforce and strategies to address service capacity relative to demand.

At the same time, changes in population needs relative to GP capacity might help explain why GP exits are perceived as a growing problem. Figure 4 shows the number of inhabitants per GP, which has declined over the period. On the surface this suggests improved capacity. However, reports show that the average size of patient lists among contracted GPs has declined sharply in recent years [21]. This reflects that many inhabitants are placed on lists without an assigned GP. As a result, shortages are more visible today than before, so even normal retirements might have a greater impact and reinforce the perception that more GPs are leaving.

**Figure 4.**
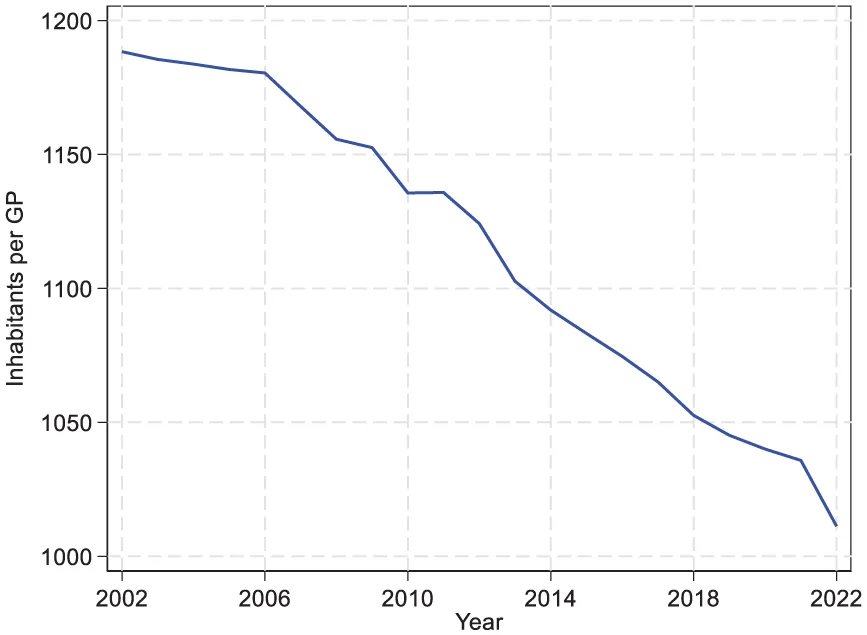
Inhabitants per general practitioner (GP) in Norwegian general practice 2002–2022.

Finally, this study highlights the value of administrative data for workforce planning. Such data can inform recruitment strategies and help identify emerging capacity constraints, not only by tracking exits but also by capturing trends in hours worked and age distribution across the GP workforce. Although this study focuses on Norway, the issues it addresses are not unique. The use of administrative data to inform workforce planning might be relevant in other countries facing similar concerns.

## Supplemental Material

sj-docx-1-sjp-10.1177_14034948251394639 – Supplemental material for Career duration in general practice: Evidence from Two Decades of Norwegian Registry Data Supplemental material, sj-docx-1-sjp-10.1177_14034948251394639 for Career duration in general practice: Evidence from Two Decades of Norwegian Registry Data by Øyvind Snilsberg, Johannes B. Tyrihjell and Geir Godager in Scandinavian Journal of Public Health
