# Supplementary material for: Career duration in general practice: Evidence from Two Decades of Norwegian Registry Data
Source: Scand J Public Health. 2025 Dec 2;54(8):826–31. doi: 10.1177/14034948251394639 (PMC13710708; doi:10.1177/14034948251394639)
Supplement: Supplementary file 1 [file sj-docx-1-sjp-10.1177_14034948251394639.docx]

Table S1 Exit rates in Norwegian general practice 2002–2022

| Truncated age | Exit rate 2002 [95 CI] | Exit rate 2003 [95 CI] | Exit rate 2004 [95 CI] | Exit rate 2005 [95 CI] | Exit rate 2006 [95 CI] | Exit rate 2007 [95 CI] | Exit rate 2008 [95 CI] |
| --- | --- | --- | --- | --- | --- | --- | --- |
| 28 | 0.286 [0.000, 0.333] | 0.095 [0.000, 0.333] | 0.037 [0.000, 0.296] | 0.056 [0.000, 0.333] | 0.040 [0.000, 0.280] | 0.038 [0.000, 0.308] | 0.037 [0.000, 0.296] |
| 29 | 0.075 [0.000, 0.225] | 0.056 [0.000, 0.333] | 0.120 [0.000, 0.280] | 0.138 [0.000, 0.241] | 0.167 [0.000, 0.267] | 0.111 [0.000, 0.259] | 0.031 [0.000, 0.250] |
| 30 | 0.103 [0.000, 0.207] | 0.052 [0.000, 0.207] | 0.081 [0.000, 0.243] | 0.091 [0.000, 0.227] | 0.038 [0.000, 0.208] | 0.023 [0.000, 0.227] | 0.043 [0.000, 0.217] |
| 31 | 0.094 [0.000, 0.172] | 0.088 [0.000, 0.176] | 0.104 [0.000, 0.169] | 0.059 [0.000, 0.196] | 0.089 [0.000, 0.179] | 0.055 [0.000, 0.164] | 0.032 [0.000, 0.177] |
| 32 | 0.039 [0.000, 0.169] | 0.067 [0.000, 0.173] | 0.107 [0.000, 0.167] | 0.060 [0.000, 0.167] | 0.059 [0.000, 0.176] | 0.015 [0.000, 0.179] | 0.070 [0.000, 0.163] |
| 33 | 0.070 [0.000, 0.140] | 0.066 [0.000, 0.143] | 0.083 [0.000, 0.143] | 0.078 [0.000, 0.144] | 0.040 [0.000, 0.140] | 0.036 [0.000, 0.143] | 0.034 [0.000, 0.146] |
| 34 | 0.039 [0.000, 0.143] | 0.085 [0.000, 0.138] | 0.032 [0.000, 0.137] | 0.065 [0.000, 0.141] | 0.053 [0.000, 0.137] | 0.054 [0.000, 0.126] | 0.050 [0.000, 0.130] |
| 35 | 0.069 [0.000, 0.153] | 0.071 [0.000, 0.141] | 0.074 [0.000, 0.137] | 0.068 [0.000, 0.136] | 0.042 [0.000, 0.137] | 0.061 [0.000, 0.131] | 0.076 [0.000, 0.126] |
| 36 | 0.074 [0.000, 0.137] | 0.068 [0.000, 0.149] | 0.070 [0.000, 0.140] | 0.050 [0.000, 0.130] | 0.057 [0.000, 0.132] | 0.037 [0.000, 0.130] | 0.034 [0.000, 0.126] |
| 37 | 0.098 [0.000, 0.130] | 0.081 [0.000, 0.121] | 0.051 [0.000, 0.139] | 0.035 [0.000, 0.128] | 0.038 [0.000, 0.125] | 0.018 [0.000, 0.119] | 0.018 [0.000, 0.125] |
| 38 | 0.083 [0.000, 0.131] | 0.054 [0.000, 0.130] | 0.061 [0.000, 0.122] | 0.100 [0.000, 0.138] | 0.031 [0.000, 0.124] | 0.064 [0.000, 0.119] | 0.009 [0.000, 0.121] |
| 39 | 0.042 [0.000, 0.109] | 0.072 [0.000, 0.120] | 0.056 [0.000, 0.124] | 0.032 [0.000, 0.116] | 0.037 [0.000, 0.123] | 0.029 [0.000, 0.117] | 0.045 [0.000, 0.109] |
| 40 | 0.037 [0.000, 0.110] | 0.050 [0.000, 0.109] | 0.013 [0.000, 0.125] | 0.046 [0.000, 0.126] | 0.011 [0.000, 0.116] | 0.061 [0.000, 0.122] | 0.010 [0.000, 0.117] |
| 41 | 0.034 [0.000, 0.111] | 0.037 [0.000, 0.110] | 0.025 [0.000, 0.110] | 0.071 [0.000, 0.131] | 0.046 [0.000, 0.126] | 0.029 [0.000, 0.117] | 0.049 [0.000, 0.122] |
| 42 | 0.066 [0.000, 0.102] | 0.017 [0.000, 0.103] | 0.055 [0.000, 0.110] | 0.066 [0.000, 0.107] | 0.050 [0.000, 0.125] | 0.045 [0.000, 0.114] | 0.000 [0.000, 0.109] |
| 43 | 0.029 [0.000, 0.114] | 0.030 [0.000, 0.106] | 0.067 [0.000, 0.108] | 0.037 [0.000, 0.112] | 0.043 [0.000, 0.111] | 0.051 [0.000, 0.127] | 0.077 [0.000, 0.121] |
| 44 | 0.035 [0.000, 0.092] | 0.029 [0.000, 0.105] | 0.016 [0.000, 0.093] | 0.024 [0.000, 0.098] | 0.038 [0.000, 0.106] | 0.042 [0.000, 0.093] | 0.026 [0.000, 0.118] |
| 45 | 0.029 [0.000, 0.101] | 0.022 [0.000, 0.101] | 0.019 [0.000, 0.106] | 0.045 [0.000, 0.098] | 0.058 [0.000, 0.099] | 0.029 [0.000, 0.107] | 0.017 [0.000, 0.103] |
| 46 | 0.036 [0.000, 0.096] | 0.036 [0.000, 0.102] | 0.029 [0.000, 0.102] | 0.029 [0.000, 0.115] | 0.038 [0.000, 0.098] | 0.034 [0.000, 0.109] | 0.038 [0.000, 0.113] |
| 47 | 0.030 [0.000, 0.078] | 0.012 [0.000, 0.080] | 0.007 [0.000, 0.082] | 0.015 [0.000, 0.081] | 0.010 [0.000, 0.088] | 0.024 [0.000, 0.087] | 0.033 [0.000, 0.083] |
| 48 | 0.000 [0.000, 0.084] | 0.030 [0.000, 0.085] | 0.037 [0.000, 0.085] | 0.044 [0.000, 0.088] | 0.022 [0.000, 0.088] | 0.049 [0.000, 0.098] | 0.024 [0.000, 0.094] |
| 49 | 0.034 [0.000, 0.079] | 0.030 [0.000, 0.083] | 0.025 [0.000, 0.087] | 0.031 [0.000, 0.088] | 0.046 [0.000, 0.092] | 0.015 [0.000, 0.088] | 0.030 [0.000, 0.100] |
| 50 | 0.020 [0.000, 0.074] | 0.025 [0.000, 0.070] | 0.018 [0.000, 0.072] | 0.006 [0.000, 0.076] | 0.026 [0.000, 0.077] | 0.008 [0.000, 0.079] | 0.007 [0.000, 0.081] |
| 51 | 0.027 [0.000, 0.074] | 0.048 [0.000, 0.075] | 0.026 [0.000, 0.066] | 0.012 [0.000, 0.073] | 0.031 [0.000, 0.075] | 0.026 [0.000, 0.072] | 0.008 [0.000, 0.078] |
| 52 | 0.006 [0.000, 0.070] | 0.014 [0.000, 0.076] | 0.007 [0.000, 0.071] | 0.026 [0.000, 0.068] | 0.024 [0.000, 0.073] | 0.032 [0.000, 0.070] | 0.033 [0.000, 0.073] |
| 53 | 0.008 [0.000, 0.084] | 0.019 [0.000, 0.070] | 0.007 [0.000, 0.077] | 0.021 [0.000, 0.078] | 0.011 [0.000, 0.069] | 0.037 [0.000, 0.074] | 0.007 [0.000, 0.072] |
| 54 | 0.017 [0.000, 0.087] | 0.034 [0.000, 0.085] | 0.032 [0.000, 0.076] | 0.042 [0.000, 0.076] | 0.007 [0.000, 0.080] | 0.027 [0.000, 0.069] | 0.019 [0.000, 0.076] |
| 55 | 0.049 [0.000, 0.087] | 0.009 [0.000, 0.087] | 0.017 [0.000, 0.087] | 0.052 [0.000, 0.078] | 0.036 [0.000, 0.080] | 0.014 [0.000, 0.080] | 0.016 [0.000, 0.071] |
| 56 | 0.055 [0.000, 0.100] | 0.061 [0.000, 0.101] | 0.035 [0.000, 0.096] | 0.018 [0.000, 0.097] | 0.028 [0.000, 0.090] | 0.008 [0.000, 0.090] | 0.066 [0.000, 0.088] |
| 57 | 0.083 [0.000, 0.104] | 0.038 [0.000, 0.095] | 0.053 [0.000, 0.106] | 0.036 [0.000, 0.099] | 0.009 [0.000, 0.099] | 0.014 [0.000, 0.085] | 0.045 [0.000, 0.091] |
| 58 | 0.062 [0.000, 0.093] | 0.022 [0.000, 0.089] | 0.039 [0.000, 0.087] | 0.011 [0.000, 0.088] | 0.046 [0.000, 0.083] | 0.009 [0.000, 0.081] | 0.042 [0.000, 0.076] |
| 59 | 0.000 [0.000, 0.109] | 0.044 [0.000, 0.088] | 0.056 [0.000, 0.089] | 0.030 [0.000, 0.091] | 0.033 [0.000, 0.098] | 0.038 [0.000, 0.085] | 0.018 [0.000, 0.082] |
| 60 | 0.073 [0.000, 0.127] | 0.031 [0.000, 0.109] | 0.046 [0.000, 0.092] | 0.047 [0.000, 0.094] | 0.041 [0.000, 0.093] | 0.011 [0.000, 0.097] | 0.029 [0.000, 0.087] |
| 61 | 0.000 [0.000, 0.128] | 0.020 [0.000, 0.118] | 0.000 [0.000, 0.109] | 0.000 [0.000, 0.096] | 0.012 [0.000, 0.099] | 0.053 [0.000, 0.096] | 0.043 [0.000, 0.097] |
| 62 | 0.000 [0.000, 0.154] | 0.021 [0.000, 0.149] | 0.040 [0.000, 0.160] | 0.016 [0.000, 0.141] | 0.024 [0.000, 0.120] | 0.049 [0.000, 0.123] | 0.079 [0.000, 0.124] |
| 63 | 0.073 [0.000, 0.171] | 0.038 [0.000, 0.154] | 0.043 [0.000, 0.174] | 0.041 [0.000, 0.163] | 0.000 [0.000, 0.156] | 0.074 [0.000, 0.136] | 0.052 [0.000, 0.143] |
| 64 | 0.086 [0.000, 0.229] | 0.154 [0.000, 0.205] | 0.000 [0.000, 0.200] | 0.068 [0.000, 0.205] | 0.021 [0.000, 0.188] | 0.062 [0.000, 0.172] | 0.067 [0.000, 0.160] |
| 65 | 0.037 [0.000, 0.296] | 0.125 [0.000, 0.281] | 0.121 [0.000, 0.273] | 0.180 [0.000, 0.240] | 0.024 [0.000, 0.244] | 0.085 [0.000, 0.234] | 0.133 [0.000, 0.217] |
| 66 | 0.056 [0.000, 0.389] | 0.222 [0.000, 0.333] | 0.107 [0.000, 0.321] | 0.200 [0.000, 0.333] | 0.146 [0.000, 0.293] | 0.100 [0.000, 0.300] | 0.182 [0.000, 0.273] |
| 67 | 0.167 [0.000, 0.750] | 0.176 [0.000, 0.647] | 0.476 [0.000, 0.619] | 0.320 [0.040, 0.600] | 0.458 [0.042, 0.625] | 0.571 [0.057, 0.543] | 0.270 [0.081, 0.541] |
| 68 | 0.000 [0.000, 0.750] | 0.200 [0.000, 0.700] | 0.143 [0.000, 0.643] | 0.545 [0.000, 0.636] | 0.176 [0.000, 0.588] | 0.077 [0.000, 0.615] | 0.133 [0.000, 0.600] |
| 69 | 0.000 [0.000, 1.000] | 0.250 [0.000, 0.750] | 0.375 [0.000, 0.750] | 0.333 [0.000, 0.667] | 0.000 [0.000, 0.800] | 0.200 [0.000, 0.600] | 0.154 [0.000, 0.615] |
| 70 | 0.200 [0.067, 0.867] | 0.375 [0.062, 0.812] | 0.375 [0.062, 0.812] | 0.667 [0.067, 0.867] | 0.385 [0.077, 0.846] | 0.385 [0.077, 0.846] | 0.429 [0.095, 0.810] |

| Truncated age | Exit rate 2009 [95 CI] | Exit rate 2010 [95 CI] | Exit rate 2011 [95 CI] | Exit rate 2012 [95 CI] | Exit rate 2013 [95 CI] | Exit rate 2014 [95 CI] | Exit rate 2015 [95 CI] |
| --- | --- | --- | --- | --- | --- | --- | --- |
| 28 | 0.100 [0.000, 0.350] | 0.059 [0.000, 0.353] | 0.190 [0.000, 0.333] | 0.069 [0.000, 0.276] | 0.000 [0.000, 0.250] | 0.111 [0.000, 0.250] | 0.075 [0.000, 0.250] |
| 29 | 0.057 [0.000, 0.229] | 0.115 [0.000, 0.269] | 0.038 [0.000, 0.269] | 0.034 [0.000, 0.241] | 0.098 [0.000, 0.220] | 0.078 [0.000, 0.196] | 0.068 [0.000, 0.205] |
| 30 | 0.070 [0.000, 0.211] | 0.039 [0.000, 0.216] | 0.048 [0.000, 0.238] | 0.058 [0.000, 0.212] | 0.053 [0.000, 0.211] | 0.031 [0.000, 0.200] | 0.135 [0.000, 0.189] |
| 31 | 0.031 [0.000, 0.172] | 0.031 [0.000, 0.156] | 0.069 [0.000, 0.167] | 0.034 [0.000, 0.190] | 0.062 [0.000, 0.163] | 0.051 [0.000, 0.165] | 0.047 [0.000, 0.165] |
| 32 | 0.039 [0.000, 0.169] | 0.061 [0.000, 0.153] | 0.069 [0.009, 0.147] | 0.019 [0.000, 0.154] | 0.060 [0.000, 0.169] | 0.064 [0.000, 0.147] | 0.081 [0.000, 0.152] |
| 33 | 0.070 [0.000, 0.140] | 0.023 [0.000, 0.149] | 0.050 [0.000, 0.126] | 0.023 [0.000, 0.123] | 0.053 [0.000, 0.129] | 0.010 [0.000, 0.135] | 0.042 [0.000, 0.127] |
| 34 | 0.065 [0.000, 0.130] | 0.071 [0.000, 0.125] | 0.058 [0.000, 0.135] | 0.060 [0.000, 0.119] | 0.046 [0.007, 0.118] | 0.020 [0.007, 0.119] | 0.032 [0.000, 0.128] |
| 35 | 0.036 [0.000, 0.127] | 0.070 [0.000, 0.130] | 0.016 [0.000, 0.121] | 0.024 [0.000, 0.119] | 0.048 [0.007, 0.116] | 0.047 [0.006, 0.112] | 0.036 [0.006, 0.114] |
| 36 | 0.073 [0.000, 0.121] | 0.071 [0.000, 0.118] | 0.025 [0.000, 0.123] | 0.022 [0.000, 0.119] | 0.042 [0.000, 0.120] | 0.032 [0.006, 0.114] | 0.078 [0.006, 0.111] |
| 37 | 0.025 [0.000, 0.115] | 0.039 [0.000, 0.118] | 0.055 [0.000, 0.117] | 0.059 [0.000, 0.111] | 0.035 [0.000, 0.112] | 0.033 [0.000, 0.106] | 0.031 [0.006, 0.104] |
| 38 | 0.025 [0.000, 0.119] | 0.076 [0.000, 0.115] | 0.031 [0.000, 0.115] | 0.045 [0.000, 0.112] | 0.051 [0.000, 0.109] | 0.033 [0.000, 0.105] | 0.032 [0.000, 0.102] |
| 39 | 0.025 [0.000, 0.108] | 0.049 [0.000, 0.107] | 0.023 [0.000, 0.105] | 0.030 [0.000, 0.106] | 0.059 [0.000, 0.103] | 0.028 [0.000, 0.099] | 0.037 [0.000, 0.099] |
| 40 | 0.082 [0.000, 0.109] | 0.025 [0.000, 0.107] | 0.024 [0.000, 0.106] | 0.015 [0.000, 0.102] | 0.036 [0.000, 0.101] | 0.058 [0.000, 0.102] | 0.034 [0.000, 0.103] |
| 41 | 0.046 [0.000, 0.110] | 0.113 [0.000, 0.113] | 0.033 [0.000, 0.107] | 0.041 [0.000, 0.107] | 0.027 [0.000, 0.103] | 0.028 [0.000, 0.106] | 0.046 [0.000, 0.107] |
| 42 | 0.025 [0.000, 0.123] | 0.028 [0.000, 0.110] | 0.029 [0.000, 0.108] | 0.032 [0.000, 0.103] | 0.055 [0.000, 0.102] | 0.014 [0.000, 0.096] | 0.014 [0.000, 0.096] |
| 43 | 0.055 [0.000, 0.109] | 0.013 [0.000, 0.125] | 0.037 [0.000, 0.111] | 0.000 [0.000, 0.110] | 0.054 [0.000, 0.101] | 0.031 [0.000, 0.102] | 0.020 [0.000, 0.099] |
| 44 | 0.034 [0.000, 0.103] | 0.028 [0.000, 0.102] | 0.060 [0.000, 0.107] | 0.037 [0.000, 0.103] | 0.058 [0.000, 0.106] | 0.008 [0.000, 0.092] | 0.055 [0.000, 0.094] |
| 45 | 0.014 [0.000, 0.122] | 0.080 [0.000, 0.115] | 0.037 [0.000, 0.111] | 0.012 [0.000, 0.118] | 0.037 [0.000, 0.110] | 0.020 [0.000, 0.111] | 0.045 [0.000, 0.097] |
| 46 | 0.017 [0.000, 0.110] | 0.027 [0.000, 0.120] | 0.000 [0.000, 0.116] | 0.045 [0.000, 0.108] | 0.054 [0.000, 0.120] | 0.046 [0.000, 0.110] | 0.079 [0.000, 0.109] |
| 47 | 0.010 [0.000, 0.095] | 0.034 [0.000, 0.085] | 0.081 [0.000, 0.108] | 0.011 [0.000, 0.103] | 0.028 [0.000, 0.092] | 0.011 [0.000, 0.096] | 0.029 [0.000, 0.095] |
| 48 | 0.008 [0.000, 0.092] | 0.038 [0.000, 0.094] | 0.017 [0.000, 0.094] | 0.014 [0.000, 0.114] | 0.012 [0.000, 0.105] | 0.027 [0.000, 0.100] | 0.064 [0.000, 0.106] |
| 49 | 0.032 [0.000, 0.095] | 0.042 [0.000, 0.093] | 0.010 [0.000, 0.097] | 0.035 [0.000, 0.096] | 0.026 [0.000, 0.118] | 0.012 [0.000, 0.105] | 0.064 [0.000, 0.100] |
| 50 | 0.010 [0.000, 0.091] | 0.024 [0.000, 0.080] | 0.026 [0.000, 0.085] | 0.009 [0.000, 0.085] | 0.061 [0.000, 0.088] | 0.067 [0.000, 0.107] | 0.023 [0.000, 0.092] |
| 51 | 0.022 [0.000, 0.082] | 0.030 [0.000, 0.090] | 0.016 [0.000, 0.081] | 0.034 [0.000, 0.085] | 0.009 [0.000, 0.085] | 0.019 [0.000, 0.083] | 0.014 [0.000, 0.097] |
| 52 | 0.000 [0.000, 0.076] | 0.053 [0.000, 0.075] | 0.010 [0.000, 0.081] | 0.065 [0.000, 0.081] | 0.017 [0.000, 0.077] | 0.009 [0.000, 0.084] | 0.019 [0.000, 0.083] |
| 53 | 0.014 [0.000, 0.075] | 0.008 [0.000, 0.075] | 0.024 [0.000, 0.079] | 0.020 [0.000, 0.092] | 0.043 [0.000, 0.078] | 0.034 [0.000, 0.078] | 0.037 [0.000, 0.084] |
| 54 | 0.007 [0.000, 0.072] | 0.028 [0.000, 0.076] | 0.007 [0.000, 0.075] | 0.016 [0.000, 0.081] | 0.031 [0.000, 0.093] | 0.035 [0.000, 0.088] | 0.035 [0.000, 0.080] |
| 55 | 0.013 [0.000, 0.078] | 0.026 [0.000, 0.077] | 0.000 [0.000, 0.078] | 0.015 [0.000, 0.083] | 0.025 [0.000, 0.083] | 0.021 [0.000, 0.095] | 0.000 [0.000, 0.088] |
| 56 | 0.033 [0.000, 0.082] | 0.026 [0.000, 0.085] | 0.040 [0.000, 0.086] | 0.028 [0.000, 0.092] | 0.023 [0.000, 0.091] | 0.059 [0.000, 0.092] | 0.011 [0.000, 0.108] |
| 57 | 0.031 [0.000, 0.094] | 0.023 [0.000, 0.079] | 0.013 [0.000, 0.087] | 0.041 [0.000, 0.090] | 0.029 [0.000, 0.086] | 0.023 [0.000, 0.093] | 0.018 [0.000, 0.097] |
| 58 | 0.016 [0.000, 0.079] | 0.008 [0.000, 0.080] | 0.029 [0.000, 0.069] | 0.027 [0.000, 0.074] | 0.007 [0.000, 0.079] | 0.051 [0.000, 0.080] | 0.000 [0.000, 0.078] |
| 59 | 0.014 [0.000, 0.079] | 0.016 [0.000, 0.079] | 0.024 [0.000, 0.079] | 0.018 [0.000, 0.071] | 0.028 [0.000, 0.076] | 0.014 [0.000, 0.078] | 0.023 [0.000, 0.084] |
| 60 | 0.028 [0.000, 0.093] | 0.057 [0.000, 0.078] | 0.024 [0.000, 0.087] | 0.024 [0.000, 0.081] | 0.012 [0.000, 0.078] | 0.036 [0.000, 0.079] | 0.007 [0.000, 0.078] |
| 61 | 0.020 [0.000, 0.090] | 0.029 [0.000, 0.095] | 0.037 [0.000, 0.081] | 0.064 [0.000, 0.088] | 0.025 [0.000, 0.092] | 0.030 [0.000, 0.079] | 0.022 [0.000, 0.081] |
| 62 | 0.033 [0.000, 0.120] | 0.041 [0.000, 0.112] | 0.029 [0.000, 0.118] | 0.069 [0.000, 0.107] | 0.060 [0.000, 0.111] | 0.034 [0.000, 0.111] | 0.043 [0.000, 0.099] |
| 63 | 0.061 [0.000, 0.134] | 0.034 [0.000, 0.135] | 0.043 [0.000, 0.128] | 0.040 [0.000, 0.121] | 0.057 [0.000, 0.115] | 0.036 [0.000, 0.118] | 0.044 [0.000, 0.123] |
| 64 | 0.055 [0.000, 0.164] | 0.076 [0.000, 0.165] | 0.035 [0.000, 0.151] | 0.044 [0.000, 0.156] | 0.053 [0.000, 0.147] | 0.043 [0.000, 0.139] | 0.028 [0.000, 0.142] |
| 65 | 0.043 [0.000, 0.214] | 0.029 [0.000, 0.214] | 0.110 [0.000, 0.205] | 0.072 [0.000, 0.193] | 0.116 [0.012, 0.198] | 0.078 [0.011, 0.189] | 0.082 [0.009, 0.182] |
| 66 | 0.077 [0.000, 0.269] | 0.090 [0.015, 0.239] | 0.147 [0.015, 0.250] | 0.123 [0.000, 0.246] | 0.130 [0.013, 0.234] | 0.105 [0.013, 0.237] | 0.084 [0.012, 0.229] |
| 67 | 0.222 [0.083, 0.556] | 0.396 [0.104, 0.521] | 0.311 [0.115, 0.492] | 0.310 [0.103, 0.483] | 0.404 [0.105, 0.491] | 0.269 [0.119, 0.478] | 0.206 [0.118, 0.471] |
| 68 | 0.286 [0.000, 0.500] | 0.143 [0.000, 0.500] | 0.138 [0.000, 0.483] | 0.381 [0.048, 0.429] | 0.200 [0.025, 0.450] | 0.147 [0.029, 0.471] | 0.306 [0.041, 0.429] |
| 69 | 0.154 [0.000, 0.615] | 0.200 [0.000, 0.550] | 0.208 [0.000, 0.500] | 0.160 [0.000, 0.520] | 0.192 [0.000, 0.500] | 0.125 [0.031, 0.469] | 0.103 [0.000, 0.483] |
| 70 | 0.435 [0.130, 0.783] | 0.583 [0.125, 0.792] | 0.538 [0.154, 0.769] | 0.452 [0.161, 0.742] | 0.421 [0.184, 0.711] | 0.488 [0.209, 0.698] | 0.480 [0.220, 0.680] |

| Truncated age | Exit rate 2016 [95 CI] | Exit rate 2017 [95 CI] | Exit rate 2018 [95 CI] | Exit rate 2019 [95 CI] | Exit rate 2020 [95 CI] | Exit rate 2021 [95 CI] | Exit rate 2022 [95 CI] |
| --- | --- | --- | --- | --- | --- | --- | --- |
| 28 | 0.000 [0.000, 0.233] | 0.083 [0.000, 0.229] | 0.137 [0.000, 0.216] | 0.068 [0.000, 0.227] | 0.118 [0.000, 0.265] | 0.065 [0.000, 0.258] | 0.071 [0.000, 0.286] |
| 29 | 0.048 [0.000, 0.214] | 0.019 [0.000, 0.212] | 0.073 [0.000, 0.200] | 0.000 [0.000, 0.193] | 0.049 [0.000, 0.197] | 0.081 [0.000, 0.243] | 0.069 [0.000, 0.190] |
| 30 | 0.045 [0.000, 0.197] | 0.108 [0.000, 0.189] | 0.078 [0.000, 0.182] | 0.057 [0.000, 0.186] | 0.109 [0.000, 0.168] | 0.115 [0.000, 0.172] | 0.016 [0.000, 0.197] |
| 31 | 0.053 [0.000, 0.147] | 0.067 [0.000, 0.157] | 0.098 [0.000, 0.152] | 0.036 [0.000, 0.145] | 0.072 [0.000, 0.155] | 0.054 [0.000, 0.143] | 0.058 [0.008, 0.142] |
| 32 | 0.047 [0.000, 0.150] | 0.089 [0.000, 0.152] | 0.083 [0.000, 0.156] | 0.054 [0.000, 0.152] | 0.109 [0.008, 0.147] | 0.049 [0.008, 0.148] | 0.073 [0.013, 0.140] |
| 33 | 0.102 [0.000, 0.139] | 0.071 [0.000, 0.135] | 0.047 [0.000, 0.133] | 0.033 [0.000, 0.133] | 0.033 [0.000, 0.131] | 0.094 [0.007, 0.130] | 0.090 [0.006, 0.123] |
| 34 | 0.062 [0.000, 0.125] | 0.041 [0.000, 0.130] | 0.042 [0.007, 0.127] | 0.042 [0.007, 0.127] | 0.055 [0.007, 0.124] | 0.058 [0.007, 0.124] | 0.106 [0.006, 0.119] |
| 35 | 0.049 [0.000, 0.119] | 0.052 [0.000, 0.119] | 0.081 [0.000, 0.119] | 0.031 [0.006, 0.113] | 0.043 [0.006, 0.111] | 0.044 [0.006, 0.112] | 0.057 [0.006, 0.115] |
| 36 | 0.039 [0.006, 0.106] | 0.024 [0.006, 0.107] | 0.068 [0.000, 0.115] | 0.055 [0.000, 0.116] | 0.040 [0.006, 0.109] | 0.050 [0.006, 0.106] | 0.034 [0.006, 0.108] |
| 37 | 0.028 [0.006, 0.099] | 0.082 [0.005, 0.098] | 0.049 [0.005, 0.099] | 0.070 [0.000, 0.108] | 0.044 [0.000, 0.107] | 0.032 [0.005, 0.101] | 0.030 [0.005, 0.096] |
| 38 | 0.047 [0.006, 0.105] | 0.016 [0.005, 0.102] | 0.058 [0.005, 0.099] | 0.044 [0.005, 0.104] | 0.038 [0.000, 0.108] | 0.031 [0.006, 0.104] | 0.073 [0.005, 0.098] |
| 39 | 0.036 [0.000, 0.095] | 0.029 [0.000, 0.097] | 0.040 [0.005, 0.090] | 0.060 [0.005, 0.090] | 0.032 [0.005, 0.091] | 0.036 [0.000, 0.096] | 0.045 [0.000, 0.095] |
| 40 | 0.037 [0.000, 0.099] | 0.073 [0.000, 0.096] | 0.038 [0.005, 0.092] | 0.044 [0.005, 0.092] | 0.041 [0.005, 0.092] | 0.042 [0.005, 0.094] | 0.052 [0.000, 0.099] |
| 41 | 0.034 [0.000, 0.102] | 0.012 [0.000, 0.098] | 0.052 [0.000, 0.098] | 0.043 [0.005, 0.098] | 0.029 [0.005, 0.091] | 0.056 [0.005, 0.092] | 0.046 [0.005, 0.092] |
| 42 | 0.059 [0.000, 0.104] | 0.041 [0.000, 0.101] | 0.023 [0.000, 0.094] | 0.017 [0.000, 0.093] | 0.032 [0.000, 0.091] | 0.073 [0.005, 0.087] | 0.049 [0.005, 0.088] |
| 43 | 0.007 [0.000, 0.099] | 0.030 [0.000, 0.105] | 0.040 [0.000, 0.101] | 0.053 [0.000, 0.094] | 0.040 [0.000, 0.092] | 0.059 [0.005, 0.090] | 0.045 [0.005, 0.091] |
| 44 | 0.026 [0.000, 0.084] | 0.032 [0.000, 0.089] | 0.037 [0.000, 0.088] | 0.020 [0.000, 0.088] | 0.037 [0.000, 0.086] | 0.029 [0.000, 0.081] | 0.027 [0.000, 0.080] |
| 45 | 0.033 [0.000, 0.106] | 0.026 [0.000, 0.097] | 0.045 [0.000, 0.096] | 0.037 [0.000, 0.097] | 0.053 [0.000, 0.093] | 0.056 [0.000, 0.093] | 0.041 [0.000, 0.094] |
| 46 | 0.047 [0.000, 0.109] | 0.048 [0.000, 0.104] | 0.032 [0.000, 0.097] | 0.053 [0.000, 0.099] | 0.037 [0.000, 0.104] | 0.034 [0.000, 0.103] | 0.058 [0.000, 0.096] |
| 47 | 0.043 [0.000, 0.096] | 0.031 [0.000, 0.085] | 0.042 [0.000, 0.092] | 0.045 [0.000, 0.084] | 0.041 [0.000, 0.081] | 0.030 [0.000, 0.089] | 0.056 [0.000, 0.083] |
| 48 | 0.047 [0.000, 0.103] | 0.043 [0.000, 0.106] | 0.032 [0.000, 0.095] | 0.025 [0.000, 0.100] | 0.067 [0.000, 0.087] | 0.035 [0.000, 0.090] | 0.061 [0.000, 0.091] |
| 49 | 0.032 [0.000, 0.108] | 0.059 [0.000, 0.098] | 0.021 [0.000, 0.106] | 0.023 [0.000, 0.093] | 0.025 [0.000, 0.091] | 0.042 [0.000, 0.091] | 0.035 [0.000, 0.092] |
| 50 | 0.037 [0.000, 0.093] | 0.033 [0.000, 0.100] | 0.030 [0.000, 0.091] | 0.062 [0.000, 0.093] | 0.039 [0.000, 0.086] | 0.017 [0.000, 0.084] | 0.050 [0.000, 0.078] |
| 51 | 0.023 [0.000, 0.103] | 0.019 [0.000, 0.093] | 0.046 [0.000, 0.103] | 0.040 [0.000, 0.090] | 0.033 [0.000, 0.098] | 0.024 [0.000, 0.087] | 0.075 [0.000, 0.083] |
| 52 | 0.028 [0.000, 0.097] | 0.035 [0.000, 0.093] | 0.037 [0.000, 0.084] | 0.012 [0.000, 0.093] | 0.031 [0.000, 0.093] | 0.011 [0.000, 0.089] | 0.048 [0.000, 0.079] |
| 53 | 0.065 [0.000, 0.084] | 0.014 [0.000, 0.097] | 0.024 [0.000, 0.094] | 0.075 [0.000, 0.085] | 0.011 [0.000, 0.090] | 0.031 [0.000, 0.094] | 0.041 [0.000, 0.093] |
| 54 | 0.039 [0.000, 0.087] | 0.010 [0.000, 0.090] | 0.014 [0.000, 0.096] | 0.059 [0.000, 0.094] | 0.030 [0.000, 0.091] | 0.022 [0.000, 0.089] | 0.022 [0.000, 0.086] |
| 55 | 0.054 [0.000, 0.090] | 0.050 [0.000, 0.089] | 0.079 [0.000, 0.089] | 0.040 [0.000, 0.107] | 0.012 [0.000, 0.099] | 0.021 [0.000, 0.094] | 0.033 [0.000, 0.099] |
| 56 | 0.044 [0.000, 0.097] | 0.028 [0.000, 0.101] | 0.031 [0.000, 0.102] | 0.000 [0.000, 0.105] | 0.042 [0.000, 0.111] | 0.012 [0.000, 0.108] | 0.021 [0.000, 0.105] |
| 57 | 0.032 [0.000, 0.106] | 0.036 [0.000, 0.100] | 0.046 [0.000, 0.101] | 0.021 [0.000, 0.105] | 0.031 [0.000, 0.103] | 0.014 [0.000, 0.113] | 0.036 [0.000, 0.108] |
| 58 | 0.027 [0.000, 0.080] | 0.011 [0.000, 0.088] | 0.028 [0.000, 0.084] | 0.019 [0.000, 0.086] | 0.043 [0.000, 0.086] | 0.000 [0.000, 0.095] | 0.027 [0.000, 0.096] |
| 59 | 0.023 [0.000, 0.078] | 0.000 [0.000, 0.090] | 0.022 [0.000, 0.099] | 0.048 [0.000, 0.086] | 0.039 [0.000, 0.087] | 0.045 [0.000, 0.090] | 0.031 [0.000, 0.093] |
| 60 | 0.008 [0.000, 0.085] | 0.032 [0.000, 0.088] | 0.036 [0.000, 0.090] | 0.011 [0.000, 0.099] | 0.000 [0.000, 0.089] | 0.020 [0.000, 0.090] | 0.057 [0.000, 0.103] |
| 61 | 0.028 [0.000, 0.085] | 0.031 [0.000, 0.086] | 0.017 [0.000, 0.091] | 0.037 [0.000, 0.092] | 0.033 [0.000, 0.100] | 0.020 [0.000, 0.099] | 0.071 [0.000, 0.101] |
| 62 | 0.038 [0.000, 0.105] | 0.044 [0.000, 0.102] | 0.040 [0.000, 0.105] | 0.034 [0.000, 0.109] | 0.010 [0.000, 0.114] | 0.045 [0.000, 0.124] | 0.030 [0.000, 0.111] |
| 63 | 0.065 [0.000, 0.110] | 0.031 [0.000, 0.117] | 0.053 [0.000, 0.115] | 0.025 [0.000, 0.118] | 0.052 [0.000, 0.121] | 0.038 [0.000, 0.125] | 0.046 [0.000, 0.126] |
| 64 | 0.082 [0.000, 0.136] | 0.069 [0.007, 0.131] | 0.032 [0.000, 0.137] | 0.097 [0.000, 0.137] | 0.060 [0.000, 0.138] | 0.073 [0.000, 0.138] | 0.030 [0.000, 0.149] |
| 65 | 0.117 [0.010, 0.184] | 0.059 [0.010, 0.188] | 0.059 [0.015, 0.170] | 0.099 [0.017, 0.174] | 0.044 [0.018, 0.177] | 0.109 [0.009, 0.182] | 0.078 [0.010, 0.184] |
| 66 | 0.139 [0.020, 0.218] | 0.066 [0.022, 0.220] | 0.074 [0.021, 0.221] | 0.087 [0.031, 0.205] | 0.092 [0.028, 0.211] | 0.074 [0.028, 0.213] | 0.102 [0.020, 0.214] |
| 67 | 0.250 [0.132, 0.461] | 0.264 [0.138, 0.448] | 0.282 [0.141, 0.447] | 0.216 [0.136, 0.443] | 0.241 [0.155, 0.422] | 0.270 [0.150, 0.440] | 0.250 [0.150, 0.440] |
| 68 | 0.204 [0.056, 0.407] | 0.158 [0.053, 0.404] | 0.234 [0.062, 0.391] | 0.213 [0.066, 0.393] | 0.174 [0.072, 0.391] | 0.239 [0.091, 0.364] | 0.233 [0.068, 0.384] |
| 69 | 0.143 [0.029, 0.457] | 0.209 [0.047, 0.442] | 0.146 [0.042, 0.438] | 0.327 [0.061, 0.429] | 0.292 [0.042, 0.438] | 0.333 [0.070, 0.404] | 0.250 [0.074, 0.397] |
| 70 | 0.423 [0.231, 0.673] | 0.550 [0.233, 0.650] | 0.459 [0.246, 0.656] | 0.473 [0.257, 0.635] | 0.361 [0.250, 0.625] | 0.400 [0.262, 0.625] | 0.386 [0.273, 0.614] |

Figure S1 Exit rates in Norwegian General Practice 2002–2022

Figure S2 GP age groups in Norwegian general practice 2002–2022
